# A Dopa Decarboxylase Modulating the Immune Response of Scallop *Chlamys farreri*

**DOI:** 10.1371/journal.pone.0018596

**Published:** 2011-04-13

**Authors:** Zhi Zhou, Jialong Yang, Lingling Wang, Huan Zhang, Yang Gao, Xiaowei Shi, Mengqiang Wang, Pengfei Kong, Limei Qiu, Linsheng Song

**Affiliations:** 1 Key Laboratory of Experimental Marine Biology, Institute of Oceanology, Chinese Academy of Sciences, Qingdao, China; 2 Graduate School, Chinese Academy of Sciences, Beijing, China; University of Canterbury, New Zealand

## Abstract

**Background:**

Dopa decarboxylase (DDC) is a pyridoxal 5-phosphate (PLP)-dependent enzyme that catalyzes the decarboxylation of L-Dopa to dopamine, and involved in complex neuroendocrine-immune regulatory network. The function for DDC in the immunomodulation remains unclear in invertebrate.

**Methodology:**

The full-length cDNA encoding DDC (designated CfDDC) was cloned from mollusc scallop *Chlamys farreri*. It contained an open reading frame encoding a polypeptide of 560 amino acids. The CfDDC mRNA transcripts could be detected in all the tested tissues, including the immune tissues haemocytes and hepatopancreas. After scallops were treated with LPS stimulation, the mRNA expression level of CfDDC in haemocytes increased significantly (5.5-fold, P<0.05) at 3 h and reached the peak at 12 h (9.8-fold, P<0.05), and then recovered to the baseline level. The recombinant protein of CfDDC (rCfDDC) was expressed in *Escherichia coli* BL21 (DE3)-Transetta, and 1 mg rCfDDC could catalyze the production of 1.651±0.22 ng dopamine within 1 h *in vitro*. When the haemocytes were incubated with rCfDDC-coated agarose beads, the haemocyte encapsulation to the beads was increased significantly from 70% at 6 h to 93% at 24 h *in vitro* in comparison with that in the control (23% at 6 h to 25% at 24 h), and the increased haemocyte encapsulation was repressed by the addition of rCfDDC antibody (which is acquired via immunization 6-week old rats with rCfDDC). After the injection of DDC inhibitor methyldopa, the ROS level in haemocytes of scallops was decreased significantly to 0.41-fold (P<0.05) of blank group at 12 h and 0.47-fold (P<0.05) at 24 h, respectively.

**Conclusions:**

These results collectively suggested that CfDDC, as a homologue of DDC in scallop, modulated the immune responses such as haemocytes encapsulation as well as the ROS level through its catalytic activity, functioning as an indispensable immunomodulating enzyme in the neuroendocrine-immune regulatory network of mollusc.

## Introduction

L-DOPA decarboxylase (DDC) is a pyridoxal 5-phosphate (PLP)-dependent enzyme that catalyses the decarboxylation of L-Dopa to dopamine, one of the important catecholamines present in a wide variety of animals. DDC is also referred as aromatic L-amino acid decarboxylase (AADC) in mammalian to decarboxylate aromatic L-amino acids [1], [2], [3]. By catalyzing the reaction to produce dopamine, DDC is involved in many important metabolic processes and plays a central role in the complex neuroendocrine-immune regulatory network [4]. In vertebrate, the structure and distribution of DDC is essential for functioning. DDC is found to be a homodimer *in vivo*, and each subunit can bind one PLP molecule [5], [6]. PLP serves as the cofactor of DDC which binds PLP-binding motif in the conserved Pyridoxal_deC domain to mediate the positive reaction [7]. Human DDC distributes in all catecholaminergic neurons of the central and peripheral nervous system [8], and it has been also detected in some peripheral organs such as the liver, adrenal gland and leukocytes of rat and human [9], [10]. In invertebrate, the studies about DDC are mainly focused on the identification and characterization from insects, such as *Pseudaletia separate*, *Aedes aegypti* and *Drosophila* [11], [12], [13], and the mRNA expression of DDCs is also detected in both the central nervous system and the immunocytes [14]. In contrast with mammal and insect, relatively little is known about DDC from other animals, especially in those more primitive animals than insects.

Dopamine is a vital bidirectional signal molecule in the communication between the neuroendocrine and immune system, which acts as not only neurotransmitter and hormone in neuroendocrine system but also immunomodulator in immune system [15], [16]. DDC is responsible for the conversion of L-Dopa to dopamine, and functions in the neuroendocrine-immune regulatory network. In vertebrate, DDCs are implicated in hypothalamus-pituitary-adrenocortical (HPA) systems, sympathetic nervous system and immune system [17], [18], [19], [20] to modulate the immune response against various pathogenic microorganisms [10], [15]. Recent available evidences have demonstrated the possible immunomodulation of DDC in invertebrate. For instance, Surface-associated DDC modulates some cellular immune response such as phagocytosis, nodulation in medfly haemocytes by the melanization reaction with the involvement of phenol oxidase [21], [22], [23]. And its end production dopamine depresses the immunity of shrimp *penaeus monodon* and freshwater giant prawn *macrobrachium rosenbergii* through modulating phenoloxidase activity, reactive oxygen species (ROS), and superoxide dismutase activities [24], [25]. Although there have been some studies on the function of DDCs from mammals and insects, cognition of DDCs from mollusc in the immunomodulation is found to be quite meagre.

The scallop *Chlamys farreri* is a dioecious bivalve native to the coast of China, Korea and Japan, and contributes weightily to the aquaculture industry of northern China. In recent years, the outbreak of disease has resulted in severe mortality of scallops. Neuroendocrine regulation of immune defense system makes a major contribution to the accomplishment of immune response and the maintenance of the homeostasis during infection [26]. Investigation of dopamine metabolism gene could help further knowledge of neuroendocrine modulation to the innate immune system, and provide new insights into the disease control of the scallop. The purposes of this study were (1) to clone the full-length cDNA of DDC from *C. farreri*, (2) to detect its mRNA distribution in different tissues and the temporal expression in haemocytes after LPS stimulation, (3) to investigate the influence of surface-associated recombinant CfDDC to the haemocyte encapsulation, (4) to survey the effect of the inhibited CfDDC activity on the ROS level in haemocytes.

## Materials and Methods

### Ethics statement

The scallops used in the present study are marine cultured animals, and all the experiments are conducted according to the regulations of local and central government. Female Wistar rats were from Qingdao institute for the control of drug products (Qingdao, China), and the animal experiments were approved by the Animal Care and Use Committee at Qingdao institute for the control of drug products with a permit number of SCXK (Shandong) 20090007, which complied with the National Institute of Health Guide for the Care and Use of Laboratory Animals.

### Scallops, tissue collection, LPS and methyldopa treatment

Healthy scallops *C. farreri* were collected from a local farm in Qingdao, Shandong Province, China, and maintained in the aerated seawater at 15–18°C for two week before processing.

For the tissue distribution analysis of CfDDC mRNA, six tissues including hepatopancreas, kidney, adductor muscle, gonad, gill and mantle from six healthy adult scallops were collected as parallel samples. Haemolymph from these six scallops was also collected from the adductor muscle and then immediately centrifuged at 800× g, 4°C for 10 min to harvest the haemocytes. All these samples were stored at −80°C after addition of 1 mL TRIzol reagent (Invitrogen) for subsequent RNA extraction.

In the LPS stimulation experiment, totally one hundred and twenty scallops were employed and divided into three groups. Fifty scallops in the first group received an injection of 50 µL phosphate buffered saline (PBS, 377 mmol L^−1^ NaCl, 2.7 mmol L^−1^ KCl, 8.09 mmol L^−1^ Na_2_HPO_4_, 1.47 mmol L^−1^ KH_2_PO_4_, pH 7.4), and were employed as control group, while another fifty scallops in the second group were employed as stimulation group which received an injection of 50 µL LPS from *Escherichia coli* 0111:B4 (Sigma-Aldrich, 0.5 mg ml^−1^ in PBS). These scallops were returned to water tanks after treatment, and 6 individuals were randomly sampled at 3, 6, 12, 24 and 48 h post-injection from the stimulation and control group. The rest twenty untreated scallops were employed as blank group, and 6 individuals were randomly sampled at 0 h. The haemolymph was collected and stored as described above.

Eighty scallops were employed for the DDC inhibitor stimulation experiment, and they were divided into three groups. Thirty scallops in the first group received an injection of 50 µL DDC inhibitor methyldopa (Sigma-Aldrich, 1.0 mmol L^−1^ in PBS) and were employed as inhibitor stimulation group, while another thirty scallops in the second group received an injection of 50 µL PBS, and were employed as control group. These scallops were returned to water tanks after treatment, and 6 individuals were randomly sampled at 6, 12 and 24 h post-injection in the inhibitor stimulation and control group. The rest twenty untreated scallops were employed as blank group, and 6 individuals were randomly sampled at 0 h. About 300 µL haemolymph was collected, and centrifuged at 800× g, 4°C for 10 min to harvest the haemocytes for the determination of ROS.

### RNA isolation and cDNA synthesis

Total RNA was isolated from the tissues of scallops using Trizol reagent (Invitrogen) according to its protocol. The first-strand synthesis was carried out based on Promega M-MLV RT Usage information using the DNase I (Promega)-treated total RNA as template and oligo(dT)-adaptor as primer (Table 1). The reaction was performed at 42°C for 1 h, terminated by heating at 95°C for 5 min. The cDNA mix was diluted to 1∶100 and stored at −80°C for subsequent SYBR Green fluorescent quantitative real-time PCR (RT-PCR).

**Table 1:** Sequences of the primers used in the experiment.

Primer	Sequence (5′-3′)	Sequence information
P1 (forward)	AAGGAGGGCACATCAAACTGG	CfDDC specific primer
P2 (forward)	CTGTGTGCAGAGAATGCTAACGAG	CfDDC specific primer
P3 (forward)	AGTCCAGCACCAGGATGAGAAG	CfDDC specific primer
P4 (reverse)	CTGACTCAGATGCCCGATATTCAC	CfDDC specific primer
P5 (reverse)	TCGTTAGCATTCTCTGCACACAG	CfDDC specific primer
P6 (reverse)	CAGGTGCCGACACTTCAAATC	CfDDC specific primer
P7 (forward)	GGCCACGCGTCGACTAGTACT_17_	Oligo(dT)-adaptor
P8 (reverse)	GGCCACGCGTCGACTAGTACG_10_	Oligo(dG)-adaptor
P9 (forward)	TGTTAGCCAGACCGTCAG	Real-time CfDDC primer
P10 (reverse)	ATTATCTCCTTCTTCGTCCTCC	Real-time CfDDC primer
P11 (forward)	CAAACAGCAGCCTCCTCGTCAT	Real-time actin primer
P12 (reverse)	CTGGGCACCTGAACCTTTCGTT	Real-time actin primer
P13 (forward)	ATGGAGAACATTCACAACCGTAGG	CfDDC recombination primer
P14 (reverse)	TTAAAAATTAAATATAGGTTCATCGAACGA	CfDDC recombination primer
M13-47	CGCCAGGGTTTTCCCAGTCACGAC	pMD18-T vector primer
RV-M	GAGCGGATAACAATTTCACACAGG	pMD18-T vector primer
T7	TAATACGACTCACTATAGGG	pEASY-E1 vector primer
T7t	GCTAGTTATTGCTCAGCGG	pEASY-E1 vector primer

### EST analysis and cloning of the full-length CfDDC cDNA

A cDNA library was constructed with the whole body of a scallop challenged by *Listonella anguillarum* as described by Wang et al. [27]. Random sequencing of the library using T3 primer yielded 6935 successful sequencing reactions. BLAST analysis of all the EST sequences revealed that one EST (no. rscag0_006949, 557 bp) was homologous to previously identified DDCs.

Six specific primers (Table 1) were designed based on the sequence of EST to clone the full-length cDNA of CfDDC by rapid amplification of cDNA ends (RACE) approach. PCR amplification to clone the 3′ end of CfDDC was carried out using sense primer P1, P2 or P3 and antisense primer Oligo(dT)-adaptor P7, while sense primer Oligo(dG)-adaptor P8 and antisense primer P4, P5 or P6 were used to get the 5′ end according to the Usage Information of 5′ RACE system (Invitrogen). All PCR amplification was performed in a PCR Thermal Cycle (TAKARA, GRADIENT PCR).

The PCR products were gel-purified and cloned into pMD18-T simple vector (Takara, Japan). After being transformed into the competent cells of *Escherichia coli* Top10F', the positive recombinants were identified through anti-ampicillin selection and PCR screening with sense vector primer RV-M and antisense vector primer M13-47 (Table 1). Three of the positive clones were sequenced on an ABI 3730 XL Automated Sequence (Applied Biosystems). The sequencing results were verified and subjected to cluster analysis.

### Sequence analysis

The homology searches of the cDNA sequence and protein sequence of CfDDC were conducted with BLAST algorithm at the National Center for Biotechnology Information (http://www.ncbi.nlm.gov/blast). The deduced amino acid sequence was analyzed with the Expert Protein Analysis System (http://www.expasy.org). SignalP 3.0 program was utilized to predict the presence and location of signal peptide, and the cleavage sites in amino acid sequence (http://www.cbs.dtu.dk/services/SignalP). The protein domain was predicted with the simple modular architecture research tool (SMART) version 5.1 (http://www.smart.emblheidelberg.de/). Multiple alignment of the CfDDC and other DDCs was performed with the ClustalW multiple alignment program (http://www.ebi.ac.uk/clustalw/) and multiple alignment show program (http://www.biosoft.net/sms/index.html). An unrooted phylogenic tree was constructed based on the deduced amino acid sequence of CfDDC and other known DDCs by the neighbor-joining (NJ) algorithm using the MEGA4.1 software (http://www.megasoftware.net). To derive the confidence value for the phylogeny analysis, bootstrap trials were replicated 1000 times.

### Real-time PCR analysis of CfDDC mRNA expression

The quantitative real-time RT-PCR was carried out in a total volume of 25 µL, containing 12.5 µL of 2×SYBR Green Master Mix (Applied Biosystems), 2 µL of the 100 times diluted cDNA, 0.5 µL of each primers (10 mmol L^−1^) and 9.5 µL of DEPC-water. A 110 bp product was amplified with the sense primer P9 and the antisense primer P10 (Table 1, designed on the basis of the full cDNA sequence of CfDDC) from cDNA template, and then sequenced to verify the PCR specificity. Two β-actin primers, the sense primer P11 and the antisense primer P12 (Table 1), were used to amplify a 94 bp fragment as an internal control to verify the successful reverse transcription and to calibrate the cDNA template. The SYBR Green RT-PCR assay was carried out in an ABI PRISM 7300 Sequence Detection System (Applied Biosystems) as described by Zhang et al [28].

### ROS assay

The determination of ROS was performed as described by Zhang [29]. The blank group was used as the reference sample, called the calibrator, and the value stood for an n-fold difference relative to the calibrator.

### Recombinant expression of CfDDC

The cDNA fragment encoding the mature peptide of CfDDC was amplified using Promega Taq polymerase with specific primers P13 and P14 (Table 1). The PCR products were gel-purified and cloned into pEASY-E1 expression vector with a His tag (Transgen, China). The recombinant plasmid (pEASY-E1-CfDDC) was transformed into Trans1-T1 phage resistant chemically competent cell (Transgen). The forward positive clones were screened by PCR using vector primer T7 and recombination primer P14 (Table 1), and further confirmed by nucleotide sequencing with vector primer T7 and T7t (Table 1). The valid recombinant plasmid (pEASY-E1-CfDDC) was extracted and transformed into *E. coli* BL21 (DE3)-Transetta (Transgen). Positive transformants were incubated in LB medium (containing 50 mg ml^−1^ ampicillin) at 37°C with shaking at 220 rpm. When the culture mediums reached OD_600_ of 0.5–0.7, the cells were incubated for 4 additional hours with the induction of IPTG at the final concentration of 1 mmol L^−1^. The recombinant protein CfDDC (designated rCfDDC) were purified by a Ni^2+^ chelating Sepharose column (GE healthcare), pooled by elution with 400 mmol L^−1^ imidazole under denatured condition (8 mol L^−1^ urea). The purified protein was refolded in gradient urea-TBS glycerol buffer (50 mmol L^−1^ Tris-HCl, 50 mmol L^−1^ NaCl, 10% glycerol, 2 mmol L^−1^ reduced glutathione, 0.2 mmol L^−1^ oxide glutathione, a gradient urea concentration of 6, 5, 4, 3, 2, 1, 0 mol L^−1^ urea in each gradient, pH 7.4; each gradient at 4°C for 12 h). Then the resultant protein were separated by reducing 15% SDS-polyacrylamide gel electrophoresis (SDS-PAGE), and visualized with Coomassie bright blue R250. The concentration of purified rCfDDC was quantified by BCA method [28]. Then the obtained protein was stored at −80°C for subsequent experiment.

### Enzyme assay of rCfDDC

The activity of rCfDDC was assayed by the method described by Fragoulis and Sekeris with slight modifications [30]. Briefly, 50 µL of rCfDDC (∼18.35 µg) was added into the reaction mixture constituted by 30 mmol L^−1^ L-Dopa and 0.4 mmol L^−1^ PLP in 500 µL PBS to start the catalytic reaction, while adding 50 µL of the final dialyzate served as negative control reaction. The reaction lasted out at 37°C for 1 h, and then was terminated by cooling the mixture to 0°C. The amount of dopamine produced was measured by dopamine ELISA kit (USCNLIFE, E0851h) according to the operation instruction. Each assay was performed at three times for statistic analysis. The enzymatic activity of rCfDDC is defined as the amount of dopamine (ng) produced by 1 mg rCfDDC in 1 h (ng h^−1^ mg^−1^).

### Antibodies preparation and Western blotting analysis

The renatured rCfDDC was dialyzed against ddH_2_O continuously and then frozen concentrated. The purified rCfDDC was immuned to 6 weeks old rats to acquire polyclonal antibody (designated anti-rCfDDC) as the method described by Cheng et al. [31].

After SDS-PAGE, the protein was electrophoretically transferred onto a 0.45 mm pore nitrocellulose membrane at 200 mA for 5 h. The membrane was blocked with PBS containing 3% BSA at 37°C for 1 h, and incubated with polyclonal antibodies anti-rCfDDC at 37°C for 1 h, following by washing three times with PBS containing 0.05% Tween-20 (PBS-T). Then the membrane was incubated with goat-anti-rat Ig-alkaline phosphatase conjugate (Southern Biotech) diluted 1∶4000 in PBS at 37°C for 1 h, and washed three times with PBS-T. Protein band was stained in freshly prepared substrate solution (100 mmol L^−1^ NaCl, 100 mmol L^−1^ Tris and 5 mmol L^−1^ MgCl_2_, pH 9.5) containing nitroblue tetrazolium (NBT, Sigma) and 5-bromo-4-chloro-3-indolyphosphate (BCIP, Sigma) for 5 min and stopped by washing with distilled water. Rats' pre-immune serum was used as negative control.

### Encapsulation *in vitro*

Encapsulation *in vitro* was performed as described by Ao [32]. Briefly, the renatured His-tagged rCfDDC or the same concentration of BSA (as a control protein) were incubated with Ni-NTA agarose beads (Qiagen) equilibrated in Tris-buffer saline (TBS, 20 mmol L^−1^ Tris-HCl, 500 mmol L^−1^ NaCl, 10 mmol L^−1^ CaCl_2_, pH 7.5) in a 1.5 mL tube with shaking at 4°C overnight. The protein-coated beads were washed with TBS four times, each for 5 min, and resuspended in TBS at 100–120 beads per microliter.

The haemolymph was withdrawn by a sterile syringe from the adductor muscle of scallops and simultaneously diluted (1∶3) in pre-cooled (4°C) anticoagulant (modified Alsever solution, 0.12 mol L^−1^ glucose, 0.03 mol L^−1^ sodium citrate, 9 mmol L^−1^ EDTA and 0.38 mol L^−1^ NaCl, pH7.2). After the haemolymph was centrifuged (800× g, 4°C for 10 min), haemocytes were suspended in 200 µL of Leibovitz L^−15^ medium (Sigma) and added to each well of an agarose-coated 48-well cell culture plate (Costar). The haemocytes were allowed to settle down for at least 10 min. Then 1 µL (100–120 beads) of the protein-coated agarose beads was added to each well, and the plate was incubated at 18°C. Haemocyte encapsulation to the agarose beads were observed after 6 and 24 h incubation, respectively. Each assay was performed in three different wells for statistic analysis.

For the antibody blockade experiment, 5 µL protein-coated beads were added in a 1.5 ml tube containing 50 µL of rat anti-rCfDDC antiserum in a total of 100 µL TBS, and the mixture was incubated at 4°C with shaking overnight. Then the beads were washed with TBS and resuspended in 5 µl TBS. Encapsulation assay *in vitro* was performed the same as described above.

### Statistical analysis

All data was given as means ± S.D. The data was subjected to one-way analysis of variance (one-way ANOVA) followed by an unpaired, two-tailed t-test. Differences were considered significant at P<0.05.

## Results

### Molecular characterization of CfDDC cDNA

**Figure 1:**
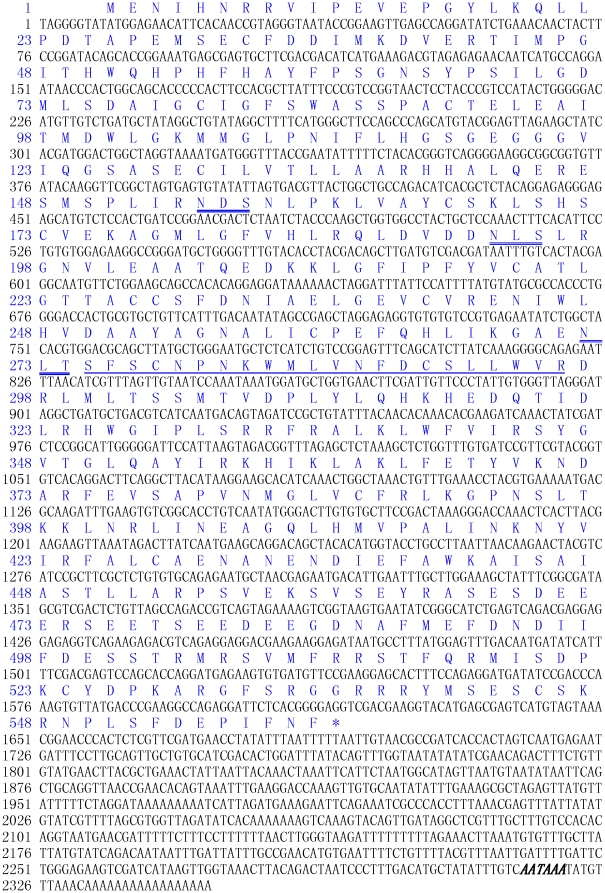
Nucleotide and deduced amino acid sequences of CfDDC. The nucleotides and amino acids are numbered along the left margin. The asterisk (*) indicates the stop codon. Polyadenylation signal is bolded and italicized. The predicted PLP-binding motif and the predicted N-glycosylation sites are underlined and double-underlined respectively.

A 2348 bp nucleotide sequence representing the complete cDNA sequence of CfDDC was obtained by overlapping EST rscag0_006949 with the amplified fragments. The sequence was deposited in GenBank under accession no. **GU131143**. The complete cDNA sequence of CfDDC and its deduced amino acids are shown in Fig. 1.

The complete sequence of CfDDC cDNA contained a 5′ untranslated region (UTR) of 9 bp, a 3′ UTR of 656 bp with a polyA tail, and an open reading frame (ORF) of 1683 bp. The ORF encoded a polypeptide of 560 amino acids with the predicted molecular weight of 63.38 kDa and theoretical isoelectric point of 6.07. There were three putative glycosylation sites within the amino acid sequence of CfDDC (Fig. 1). No signal peptide was predicted in CfDDC by SignalP software analysis. A Pyridoxal_deC domain (from Pro15 to Lys391) was identified in CfDDC by SMART program analysis, and there was a predicted PLP-binding motif (Ser275-Arg296) and a glycine-rich region (Gly115-Ser-Gly-Glu-Gly-Gly-Gly121) in the Pyridoxal_deC domain.

### Multiple sequences alignment and phylogenetic analysis

**Figure 2:**
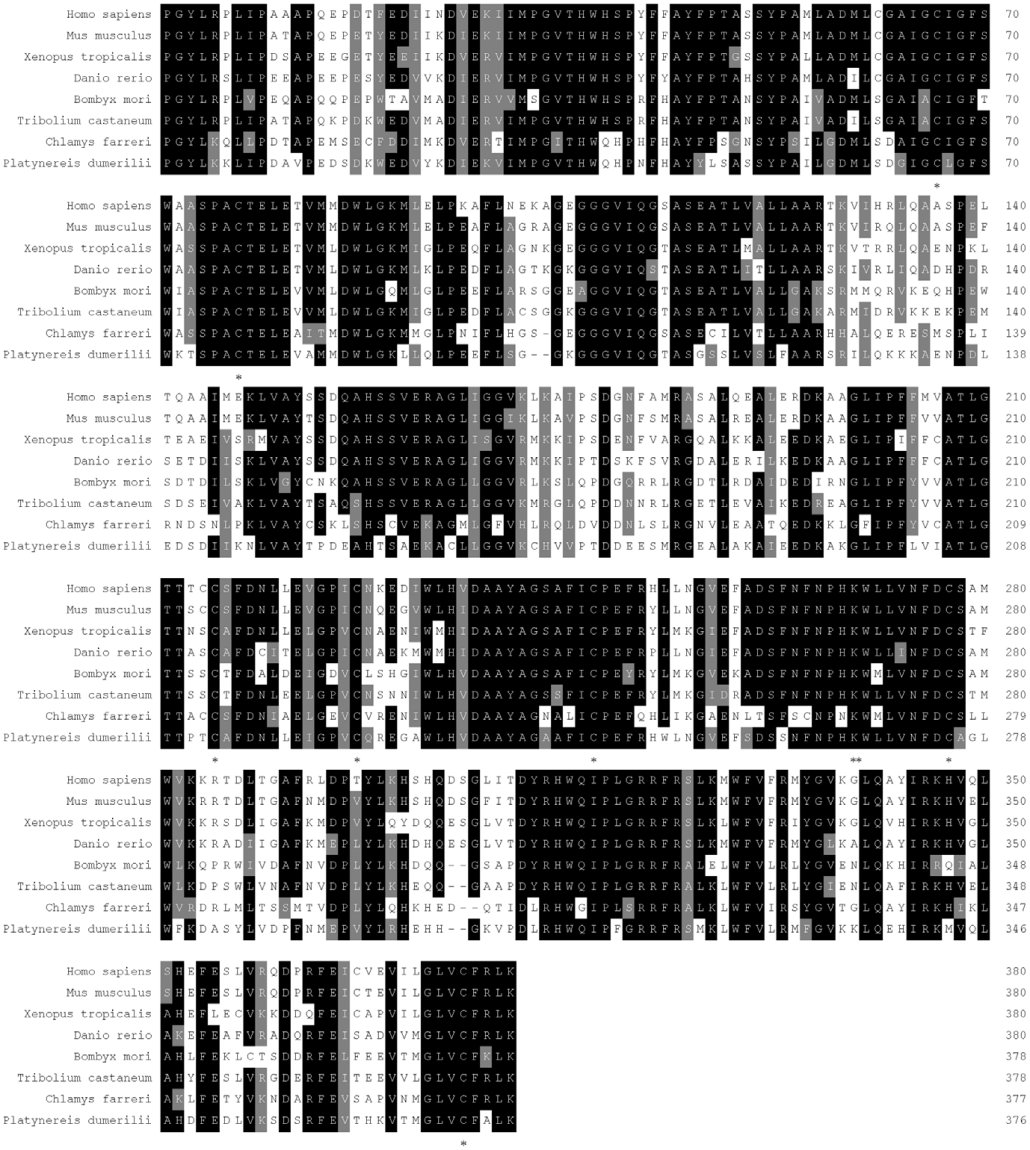
Multiple alignment of the Pyridoxal**_**deC domain of CfDDC with other DDCs deposited in GenBank. The black shadow region indicates positions where all sequences share the same amino acid residue. Similar amino acids are shaded in grey. Gaps are indicated by dashes to improve the alignment. The asterisk (*) indicates the conserved cysteine residue. The species and the GenBank accession numbers are as follows: *H. sapiens* (AAP35655), *M. musculus* (CAI23994), *X. tropicalis* (NP_001011289), *D. rerio* (AAH56292), *B. mori* (AAK48988), *T. castaneum* (ABU25222), *P. dumerilii* (CAJ38793).

The deduced amino acid sequence of CfDDC and other DDCs were aligned using the ClustalW program embedded in the program Mega 4.1 beta. CfDDC shared high similarity with other reported DDCs, such as 50.2% similarity with clamworm *Platynereis dumerilii* (CAJ38793), 52.4% with beetle *Tribolium castaneum* (ABU25222), 51.4% with silkworm *Bombyx mori* (AAK48988), 51.5% with fish *Danio rerio* (AAH56292), 51.8% with frog *Xenopus tropicalis* (NP_001011289), 52.7% with rat *Mus musculus* (CAI23994) and 52.1% with human *Homo sapiens* (AAP35655). Multiple alignments of the functional Pyridoxal_deC domain in CfDDC and other DDCs revealed seven conserved cysteine residues (Cys66, Cys77, Cys214, Cys226, Cys246, Cys276 and Cys373) (Fig. 2).

**Figure 3:**
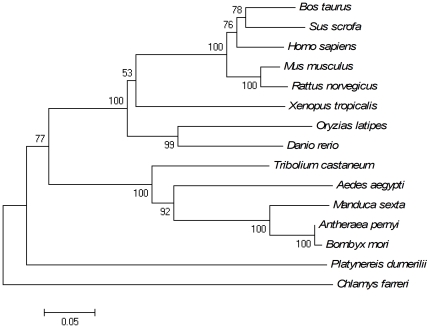
Consensus neighbour-joining tree based on the sequences of DDC from different animals. The numbers at the forks indicate the bootstrap. The protein sequences used for phylogenetic analysis include: *B. Taurus* (ABG66984), *S. scrofa* (ABO15741), *H. sapiens* (AAP35655), *M. musculus* (CAI23994), *R. norvegicus* (AAA41087), *X. tropicalis*(NP_001011289), *O. latipes* (BAH37023), *D. rerio* (AAH56292), *T. castaneum* (ABU25222), *A. aegypti* (AAC31639), *M. sexta* (AAC46604), *A. pernyi* (AAR23825), *B. mori* (AAK48988) and *P. dumerilii* (CAJ38793).

The phylogenic tree constructed based on all amino acid sequences of DDCs was shown in Fig. 3. Three distinct groups were separated in the tree. The CfDDC was first clustered with DDC from clamworm *P. dumerilii* (CAJ38793) to form the first group, and then gathered together with DDCs from arthropods in the second group, including beetle *T. castaneum* (ABU25222), mosquito *Aedes aegypti* (AAC31639), moth *Manduca sexta* (AAC46604), silkworm *B. mori* (AAK48988) and silkworm *Antheraea pernyi* (AAR23825). All the DDCs from invertebrates were clustered together and formed a sister clade to those from vertebrates, including cattle *Bos Taurus* (ABG66984), pig *Sus scrofa* (ABO15741), human *H. sapiens* (AAP35655), rat *M. musculus* (CAI23994) and mouse *Rattus norvegicus* (AAA41087), frog *X. tropicalis* (NP_001011289, fish *Oryzias latipes* (BAH37023) and fish *D. rerio* (AAH56292).

### The distribution of CfDDC mRNA in different tissues

**Figure 4:**
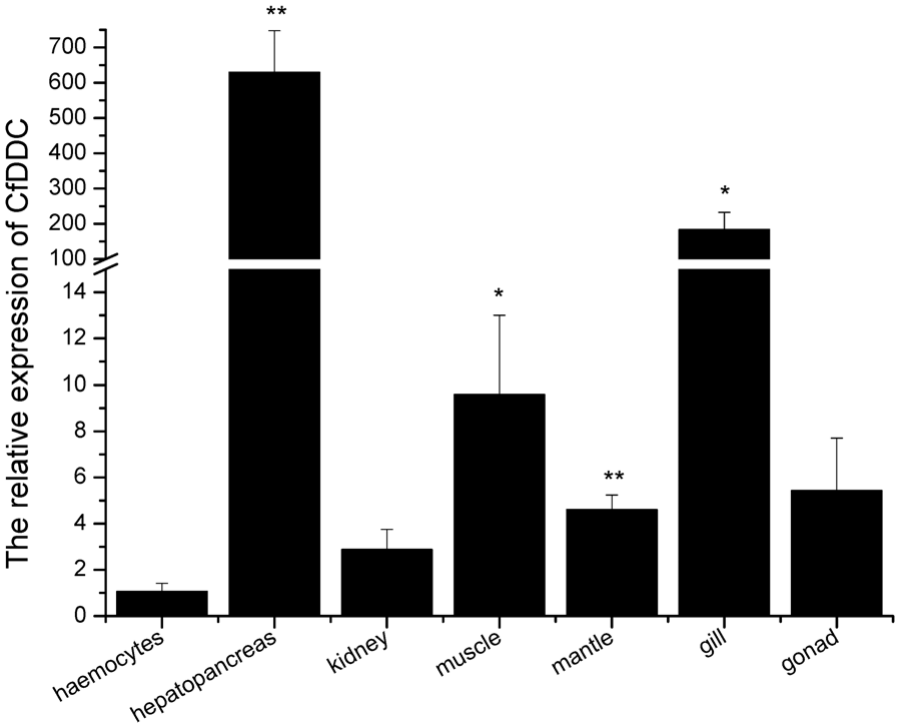
Tissues distribution of the CfDDC transcript detected by SYBR Green real-time RT-PCR. CfDDC transcript level in hepatopancreas, kidney, adductor muscle, gonad, gill and mantle of six adult scallops is normalized to that in haemocytes. Vertical bars represent the mean ± SE (N = 6).

The distribution of CfDDC mRNA in different tissues was investigated by quantitative real-time RT-PCR with β-actin as internal control. For CfDDC and β-actin gene, there was only one peak at the corresponding melting temperature in the dissociation curve analysis, indicating that the PCR was specifically amplified (data not shown). The CfDDC transcript was ubiquitously detectable in all seven tested tissues, including haemocytes, hepatopancreas, kidney, adductor muscle, mantle, gill and gonad. It was relatively abundant in hepatopancreas, adductor muscle, mantle, and gill. The highest expression of CfDDC mRNA was found in hepatopancreas, which was 629.4-fold (P<0.01) of that in haemocytes. In addition, the CfDDC expression level in adductor muscle, mantle, and gill was 9.6-fold (P<0.05), 4.6-fold (P<0.01) and 183.8-fold (P<0.05) of that in haemocytes, respectively. The CfDDC mRNA expression level in kidney and gonad was relatively low, and there were no significant differences with that in haemocytes (P>0.05) (Fig. 4).

### The temporal expression of CfDDC mRNA in haemocytes after LPS stimulation

**Figure 5:**
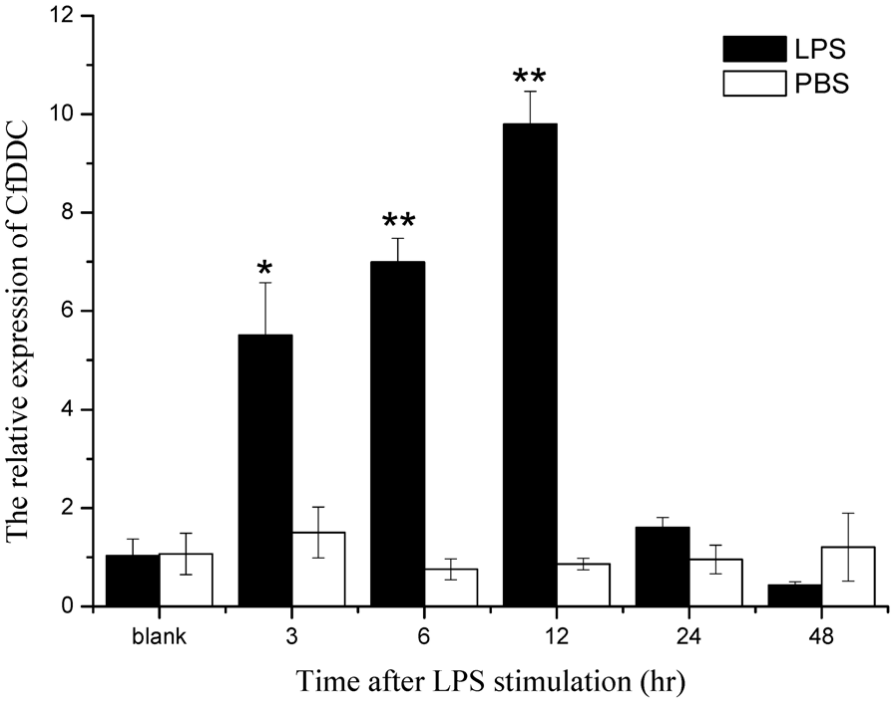
Temporal expression of CfDDC mRNA detected by real-time RT-PCR in scallop haemocytes at 3, 6, 12, 24 and 48 h after LPS stimulation. β-actin gene is used as an internal control to calibrate the cDNA template for all the samples. Each values are shown as mean ± SE (N = 6). Significant difference between challenged group and blank group is indicated by asterisks (P<0.05).

The mRNA expression of CfDDC in haemocytes of scallops after LPS stimulation was quantified by quantitative real-time RT-PCR (Fig. 5). The expression level of CfDDC mRNA began to increase significantly at 3 h (5.5-fold, P<0.05), and reached the highest expression level at 12 h (9.8-fold, P<0.01). Then it was down-regulated to 1.6-fold (P>0.05) at 24 h and 0.43-fold (P>0.05) at 48 h after LPS stimulation, respectively. Furthermore, there was no significant difference in CfDDC mRNA expression between the blank and control group during the whole experiment.

### Expression and enzyme activity of CfDDC recombination protein

**Figure 6:**
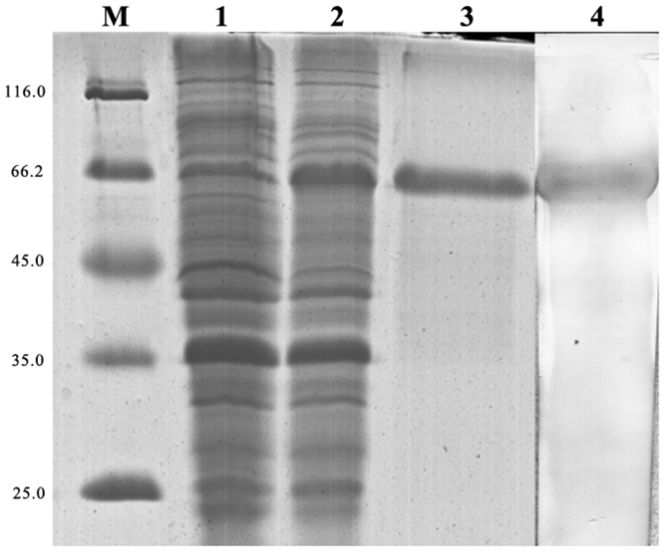
SDS-PAGE analysis of rCfDDC and the western blotting of anti-rCfDDC. Lane M: protein molecular standard (kDa). Lane 1: negative control for rCfDDC (without induction). Lane 2: IPTG induced rCfDDC. Lane 3: purified rCfDDC. Lane 4: the western blotting of anti-rCfDDC.

After IPTG induction, the whole cell lysate of *E. coli* BL21 (DE3)-Transetta with pEASY-E1-CfDDC was analyzed by SDS-PAGE. A distinct band with a molecular weight of ∼65 kDa (Fig. 6) was revealed, which was consistent with the predicted molecular mass. The concentration of the purified rCfDDC was determined to be 367 µg ml^−1^.

Dopamine ELISA kit (USCNLIFE) was used to determine the concentration of dopamine in the enzyme reaction mixture. No dopamine was detected in the control reactions, whereas there was detectable dopamine in the reactions with the addition of rCfDDC, and the enzyme activity of rCfDDC was estimated to be 1.651±0.22 ng h^−1^ mg^−1^ (N = 3).

### Western blotting of rCfDDC

The specificity of the polyclonal antibody of rCfDDC was examined by western blotting. There was only a distinct band in the nitrocellulose membrane, whose molecular weight was in accordance with that of rCfDDC (Fig. 6). Meanwhile, no distinct band was shown in the negative control.

### Haemocyte encapsulation

**Figure 7:**
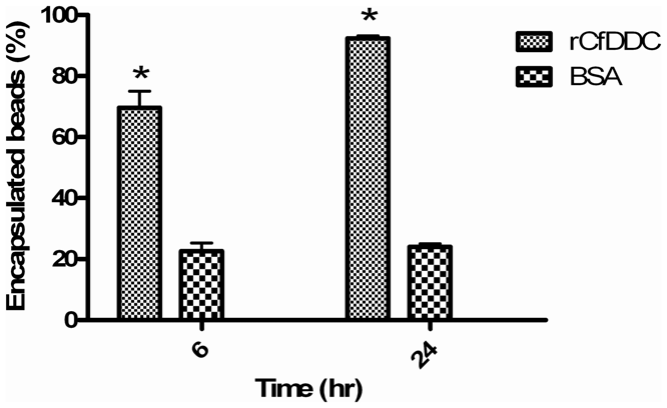
Encapsulated beads (%) of scallop haemocytes to agarose beads coated with rCfDDC and BSA at 6 h and 24 h. Vertical bars represent the mean ± SE (N = 3).

**Figure 8:**
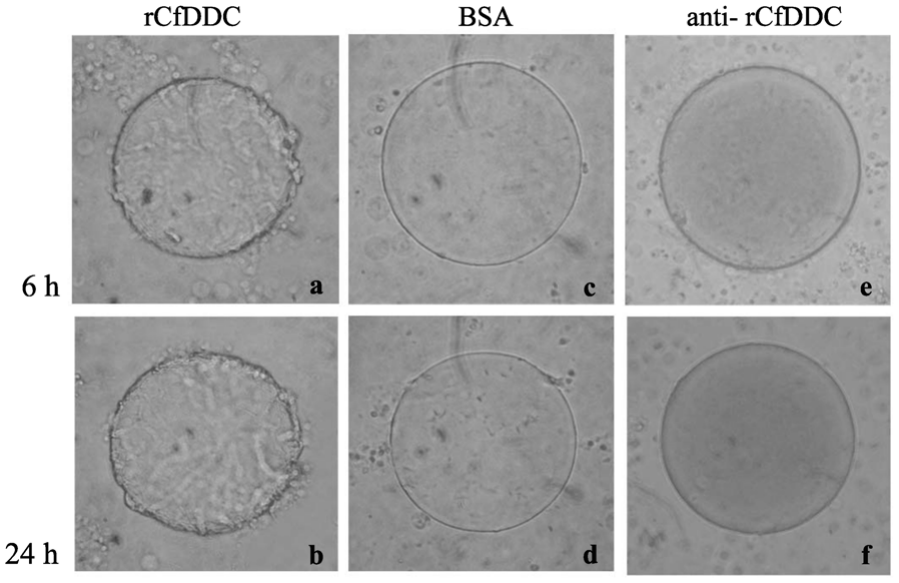
The haemocyte encapsulation promoted by rCfDDC. Agarose beads coated with rCfDDC (a, b), BSA (c, d, as a control protein), or anti-rCfDDC (e, f) were incubated with scallop haemocytes.

Haemocyte encapsulation was observed by encapsulation assay *in vitro*. When scallop haemocytes were incubated with the agarose beads, more agarose beads coated with rCfDDC were encapsulated (Fig. 7). About 70% agarose beads coated with rCfDDC were encapsulated by haemocytes of scallops within 6 h, while 23% of the beads coated with BSA were encapsulated (Fig. 8a, 8c). After 24 h of incubation, the encapsulation rate of beads coated with rCfDDC increased to 93%, but the encapsulation rate of the beads coated with BSA was only increased to 25% (Fig. 8b, 8d). However, the enhancement of rCfDDC to encapsulation was repressed by the addition of anti-rCfDDC. After adding anti-rCfDDC, the encapsulation rate of beads coated with rCfDDC decreased significantly to the 13% at 6 h and 12% at 24 h of incubation (Fig. 8e, 8f).

### ROS change in haemocytes after the injection of DDC inhibitor methyldopa

**Figure 9:**
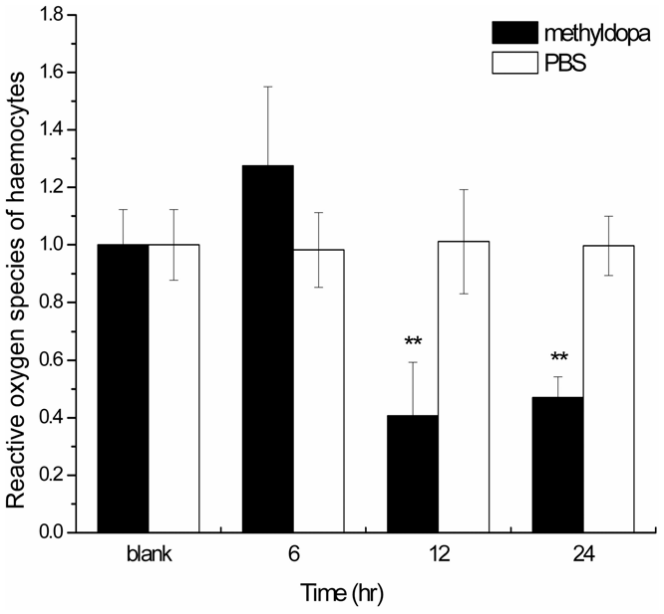
ROS level in haemocytes of scallops at 6, 12 and 24 h after the injection of DDC inhibitor methyldopa. Each values are shown as mean ± SE (N = 6). Significant difference between challenged group and blank group is indicated by asterisks (P<0.05).

The level of ROS in haemocytes of scallops after the injection of DDC inhibitor methyldopa was determined. In the inhibitor stimulation group, the level of ROS in haemocytes was decreased significantly in comparison with that in the control group (injected with PBS) (Fig. 9). It was declined to 0.41-fold of the blank group (P<0.05) at 12 h, and maintained the low level of 0.47-fold (P<0.05) at 24 h after the inhibitor stimulation.

## Discussion

DDC is a crucial metabolic enzyme responsible for catalyzing the conversion of L-Dopa to dopamine, and subsequently involved in the modulation of the immune response against pathogenic microorganism infection. In the present study, a DDC gene was identified from the scallop *C. farreri*. The full-length cDNA of CfDDC was of 2348 bp, containing a 5′ UTR of 9 bp, a 3′ UTR of 656 bp with a poly (A) tail, and an ORF of 1683 bp encoding a polypeptide of 560 amino acids. The deduced amino acids sequence of CfDDC shared high similarity (50.2–52.7%) with those of other reported DDCs. SMART program revealed that CfDDC contained a conserved Pyridoxal_deC domain, and this domain shared 70.0% similarity with that of rat *M. musculus* (CAI23994). In this functional domain, there was a PLP-binding motif and a glycine-rich region which were considered to be important for the activity of DDC [11], suggesting that CfDDC possessed the similar structure and function with previously reported DDCs. In NJ phylogenic tree, CfDDC was first clustered with DDCs from invertebrates such as clamworm and insects, and then gathered together with DDCs from vertebrates. The amino acid sequence similarity of CfDDC with other DDCs, together with its conserved functional domain and evolutionary relationship, strongly suggested it was a homologue of DDC in scallop *C. farreri*.

As an important enzyme in the neuroendocrine-immune regulatory network, DDC mainly occurred in the nervous and immune tissues in insect and chordate animals. To understand the possible function of CfDDC in the neuroendocrine-immune regulatory network of scallops, the tissue distribution of CfDDC mRNA transcript was detected by quantitative real-time RT-PCR. CfDDC mRNA transcript was detected in all the examined tissue, including haemocytes, hepatopancreas, kidney, muscle, mantle, gill and gonad. The universal distribution implied that CfDDC might be involved in the modulation of respiration, reproduction and other physiological activity of scallops by synthesizing dopamine [33], [34], [35], [36], [37], [38], [39]. It was notable that CfDDC mRNA distributed in both haemocytes and hepatopancreas, which were considered the main immune tissue of scallops [40], [41], indicating CfDDC might be implicated in the immune response of scallops and played important roles in the neuroendocrine-immune regulatory network. The higher mRNA expression of CfDDC in hepatopancreas might be partly due to the abundant distribution of catecholaminergic neurons in the hepatopancreas of scallops, which was also reported in mussels [42].

DDC in immunocytes is capable of modulating the immune response in vertebrate and insect. In mollusc, circulating haemocytes are important immunocytes responsible for the immune responses including recognition and elimination of pathogens, mainly by phagocytosis, encapsulation and nodulation, and oxidative killing [43]. To determine the response of CfDDC in scallop haemocytes to immune stimulation, the temporal expression of its mRNA was investigated in scallops stimulated by LPS. In the stimulation group, the expression level of CfDDC mRNA was increased significantly during 3–12 h in comparison with that in the control and blank group, and the peak appeared at 12 h after LPS stimulation. It suggested that LPS stimulation up-regulated the expression of CfDDC in haemocytes. And the high mRNA level might be induced by the immune mediators produced by the activated immune system. In previous studies, some immune mediators including cytokines had been reported to induce the DDC expression in insect *Pseudaletia separata* [11], [44]. The higher level of CfDDC would catalyze the decarboxylation and produce more dopamine, and these dopamine would be further converted to another catecholamine norepinephrine by dopamine beta hydroxylase in scallops [45]. These catecholamines could be released to modulate the immunological competence of scallops [4], [46], [47], [48], [49], [50], [51], and help shifting immune function into the most optimal configuration depending on the physiological context [52]. The present result indicated LPS stimulation could induce the CfDDC expression in haemocytes to produce more dopamine to modulate the immune response of scallops.

To further verify the enzyme activity of CfDDC, the recombinant protein of CfDDC (rCfDDC) was expressed in *E. coli* BL21 (DE3)-Transetta and the activity of rCfDDC was examined *in vitro* by determination of the end production dopamine in the reaction catalyze by rCfDDC. The enzyme activity of rCfDDC was 1.651±0.22 ng h^−1^ mg^−1^ (N = 3), clearly demonstrating that CfDDC had the same function to catalyze dopamine synthesis as other reported DDCs [10]. The result was in correspondence with the reports that dopamine could be determined in scallop *C. farreri* and other mollusc [53], [54], and revealed that CfDDC was involved in the complex neuroendocrine-immune regulatory network of scallops.

DDC accomplishes immunomodulation by regulating the metabolism of dopamine. For instance, DDC modulates phagocytosis, nodulation and other cellular immune response through melanization reaction with the involvement of dopamine and phenol oxidase in insect [21], [22], [55]. In the present study, the effect of surface-associated rCfDDC on haemocyte encapsulation was investigated to survey the cellular immunomodulation of CfDDC. The haemocyte encapsulation to the agarose beads coated with rCfDDC was significantly higher than that to the agarose beads coated with BSA. The antibody specific for rCfDDC significantly repressed the haemocyte encapsulation to the agarose beads coated with rCfDDC. It suggested surface-associated rCfDDC in haemocytes modulated the haemocyte encapsulation of scallops, and this modulation could be similar with that in insect implemented by a pathway in which melanin was synthesized from dopamine [14], [21]. The result indicated that the haemocyte encapsulation of scallops could be improved by the surface-associated CfDDC which was induced by the immune response. However, the downstream of DDC modulation pathway needs the involvement of other enzymes and intermediate substances, such as phenol oxidase and quinonoid [56]. Although the phenol oxidase activities in haemolymph of bivalve mollusc had been reported to be detectable [57], the molecule components of this DDC modulation pathway in mollusc as well as their respective function still needs to be further investigated.

To examine the modulation of CfDDC to the humoral immune response, DDC inhibitor methyldopa was injected to inhibit the CfDDC activity *in vivo*, and then the ROS level in haemocytes was detected in the present study. The ROS level in haemocytes was repressed significantly at 12 h (0.41-fold, P<0.05) and 24 h (0.41-fold, P<0.05) after the methyldopa injection, suggesting that CfDDC activity was necessary to keep an optimal ROS level in haemocytes. The decreased ROS level in haemocytes could result from the modulation of dopamine through the dopamine receptor or the degradation of dopamine. Dopamine could bind the dopamine receptor in the cell membrane of immunocytes to modulate the expression of antioxidases, such as superoxide dismutase [4], [25]. Furthermore, less dopamine produced by the inhibited CfDDC was transformed to hydrogen peroxide through its catabolism to decrease the ROS level in haemocytes [15]. The oxidative deamination of CfDDC to aromatic amines was also suspected to be repressed to produce less ROS [58]. Considering ROS was an important weapon of immune response against invasive pathogen [59], the decline of its level in haemocytes resulted from the inhibition of CfDDC activity by methyldopa suggested that CfDDC might depress the immune capability by terms of the change of ROS level, and consequently lead the increasing susceptibility to pathogen for scallops.

In conclusion, a DDC gene was cloned from scallop *C. farreri* and its expression in haemocytes was up-regulated by LPS stimulation. The recombinant CfDDC protein could catalyze the decarboxylation of L-Dopa to dopamine *in vitro*. And CfDDC could modulate the haemocyte encapsulation and the ROS level in haemocytes through the action of dopamine. These results indicated that CfDDC was involved in the modulation of immune response in scallops, and therefore provided evidences for CfDDC as an immunomodulating enzyme in the neuroendocrine-immune regulatory network.
